# N-glycosylation modulates the inactivation kinetics of the Kv3.4 ion channel

**DOI:** 10.1016/j.isci.2025.113409

**Published:** 2025-08-21

**Authors:** Rajnish Ranjan, Emmanuelle Logette, Mirjia Herzog, Valerie Buchillier, Enrico Scantamburlo, Henry Markram

**Affiliations:** 1 Blue Brain Project, Ecole Polytechnique Fédérale de Lausanne (EPFL), Campus Biotech, 1202 Geneva, Switzerland; 2 Laboratory of Neural Microcircuitry, Brain Mind Institute, Ecole Polytechnique Fédérale de Lausanne (EPFL), 1015 Lausanne, Switzerland

**Keywords:** Biochemistry, Cell biology

## Abstract

In our previous study, when mapping the kinetics of all 40 genetic subtypes of the voltage-gated potassium (Kv) family of ion channels, we observed significant heterogeneity in the inactivation delay of Kv3.4. Kv3.4 enables high-frequency firing in excitable cells and is linked to disorders such as Alzheimer’s disease, epilepsy, chronic pain, and cardiovascular disease. In this study, we found that N-glycosylation, a co- and post-translational process of adding glycans branches to proteins, is a key mechanism that causes heterogeneity in the inactivation delay of Kv3.4 ion channel. Additionally, we discovered that changes in glucose availability directly affect N-glycosylation and the kinetics of Kv3.4, along with other N-glycosylated Kvs, making glucose a key regulator of Kv activity and, consequently, cell excitability. We propose that disruptions in N-glycosylation of Kv3.4 ion channels may play a role in neurological disorders linked to impaired glucose metabolism.

## Highlights

• N-glycosylation modulates the inactivation delay kinetics of Kv3.4
• Glucose availability regulates Kv3.4 function via glycosylation
• Only complex-glycosylated Kv3.4 is expressed in the rodent brain
• N-glycosylation is a broader mechanism for Kv channel kinetic modulation

## Graphical abstract

**Figure:**
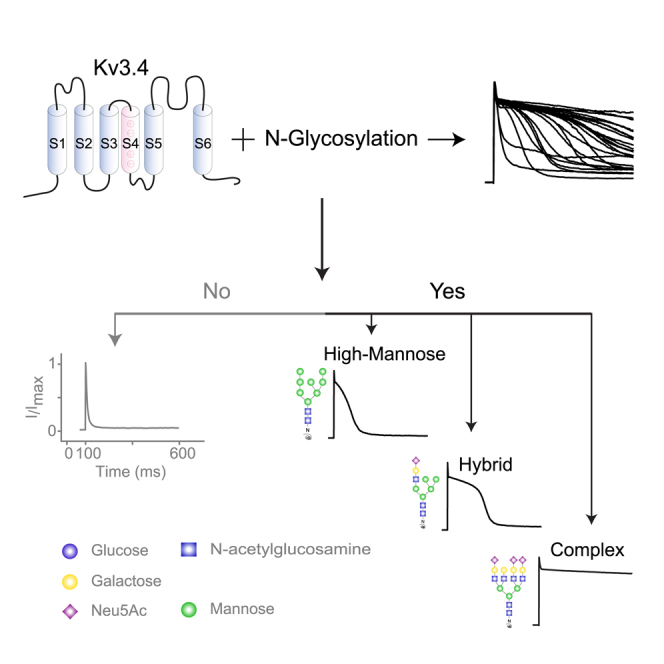

## Introduction

Voltage-gated ion channels (VGICs) are transmembrane proteins expressed in many different tissues and organs, including muscles, heart, and brain.^1^^,^^2^^,^^3^^,^^4^ They regulate the electrical excitability of cells, which is central for many physiological processes such as muscle contraction, neuronal signaling, and neurotransmitters and hormones release. Among all VGICs, voltage-gated potassium channels (Kv) represent the largest family, which is divided into 12 subfamilies, Kv1 to Kv12.^5^ Members of the Kv3 subfamily typically open at high voltages (−15 mV to +10 mV) and rapidly activate and deactivate in response to changes in voltage. These kinetic properties allow Kv3 channels to play a crucial role in rapid repolarization and in high-frequency action potential firing.^6^^,^^7^ This subfamily consists of four members (Kv3.1, Kv3.2, Kv3.3, and Kv3.4), all significantly expressed in the brain with distinct subcellular distribution and function.^8^ All Kv3 members enable fast-activating K^+^ currents, but Kv3.4 is the only member of the subfamily that also enables fast-inactivating K^+^ currents.^7^^,^^8^^,^^9^ The fast inactivation occurs through an N-type mechanism, where the first 28 amino acids of the protein form an N-terminal inactivating domain (NTID, also known as the “ball and chain” motif) that blocks the channel pore.^8^^,^^10^ The physiological role of Kv3.4 extends beyond fast repolarization to an essential regulator of axonal growth.^11^ The kinetics of Kv3.4 is also known to be modulated by hypoxia^12^ and oxidative stress^13^ and has been implicated in a range of pathologies, including cancer, cardiovascular diseases,^12^^,^^14^ seizures,^15^ and chronic pain.^16^ Moreover, Kv3.4 has also emerged as a crucial factor in several pathogenic processes of Alzheimer’s disease.^17^^,^^18^^,^^19^^,^^20^

In a previous study, we systematically characterized the kinetics of all 40 Kvs (Kv1 to Kv12 channels) and observed, in a controlled environment, that the kinetics of individual Kv channels are largely consistent at a given temperature across host cell lines and species.^21^ However, few Kv channels (Kv1.3, Kv1.5, Kv3.3, and Kv3.4) displayed striking kinetic heterogeneity, meaning that the kinetics of a single channel, recorded across cells from an isogenic expressing cell line, varied significantly.^21^ For Kv3.4, among different kinetic properties like activation, deactivation, and inactivation, the most remarkable heterogeneity was observed in the kinetics of inactivation delay. When overexpressed in Chinese hamster ovary (CHO) cells, the Kv3.4 exhibited a unique form of inactivation, which proceeded with varying degrees of delay after the pulse onset. This diversity in inactivation delay could be attributed to well-known biological mechanisms that regulate the function of a protein depending on the developmental stage, the localization, or the cellular environment. Molecular processes such as alternative promoter usage, alternative mRNA splicing, or alternative translation initiation are such processes that allow the synthesis of different protein isoforms from a single gene, with possibly different functions.^22^^,^^23^ The Kv3.4 encoding gene is subjected to such alternative splicing leading to the synthesis of several protein isoforms,^9^ and also subjected to the usage of an alternative promoter.^24^

On the other hand, hetero-multimerization or association with auxiliary subunits are other ways of modulating the gating properties of ion channels.^25^^,^^26^ Concerning Kv3.4, interaction with MinK-related peptide 2 (MiRP2), for example, affects its rate of activation and recovery from inactivation.^27^

Finally, post-translational modifications,^16^^,^^28^^,^^29^ such as phosphorylation, oxidation, ubiquitination, or glycosylation are additional mechanisms that modulate properties of ion channels.^30^ For example, phosphorylation of the NTID site of Kv3.4 converts it to a non-inactivating delayed rectifier-type channel.^10^^,^^31^ The inactivation rate of Kv3.4 is also slowed down via oxidation^32^ or by membrane lipids.^33^ Moreover, it is known that Kv3.4 is glycosylated,^8^^,^^34^ but the impact of glycosylation on its kinetic properties is not yet known.

Glycosylation, which involves the attachment of sugar moieties named glycans to lipids or proteins, is the most common post-translational modification for secreted and membrane proteins. It is also thought to be the most complex, increasing the diversity of the proteome in an incomparable way.^35^ Glycosylation has multiple functions in the cell, particularly ensuring the proper folding, trafficking, stability, or membrane localization of proteins.^36^^,^^37^ N-glycosylation, which specifically occurs on an asparagine residue of the proteins (Asn or N),^38^ is a co- and post-translational process that begins in the endoplasmic reticulum (ER) and continues in the Golgi,^36^ involving a large number of enzymatic steps^39^^,^^40^ (Figure S1). The final attached “glycan trees” are classified into three groups^41^; high-mannose (or oligo-mannose), hybrid, and complex, depending on their structure. Both hybrid and complex-glycan conjugated proteins represent the mature and functional form of glycosylated proteins,^37^^,^^42^ whereas high-mannose glycans represent the immature form. In the brain, these immature forms are atypically abundant at the surface of neurons.^43^^,^^44^

Most of the Kv channels are glycosylated,^45^ and disturbance in their N-glycosylation was shown to affect their trafficking to the cell surface, their stability, and/or their gating properties.^46^^,^^47^ In the Kv3 subfamily, all four members hold two conserved N-glycosylation sites within the first extracellular loop. Among all members, N-glycosylation of Kv3.1 is comparatively well-investigated, with studies showing that it does affect its distribution in the membrane of neuronal-derived cell lines, as well as the opening of the voltage-dependent gate.^48^^,^^49^^,^^50^^,^^51^ The N-glycosylation profile of Kv3.4 varies between brain tissue and cell liens. N-glycosylation is a complex process involving a multitude of specific enzymes, whose expression levels differ by species and tissue type. Hence, each cell line could exhibit differences in N-glycosylation profiles.^52^^,^^53^ In terms of tissue distribution, Kv3.4 is most highly expressed in the brain, followed by the kidney and urinary bladder, eye, and endocrine tissues.^54^ In healthy brain tissue, the N-glycosylation profile of Kv3.4 consists exclusively of complex-glycosylated form^34^; however, the profile in other tissues has yet to be confirmed and should be further investigated.

Glucose supplementation plays a critical role in supporting the N-glycosylation pathway. As glucose is a key precursor in the biosynthesis of nucleotide sugars required for glycan assembly. Adequate glucose availability ensures efficient production of these nucleotide sugars, thereby promoting the proper formation and maturation of N-glycans. Conversely, glucose deprivation can impair glycosylation by limiting the availability of sugar donors, leading to incomplete glycosylation, the accumulation of high-mannose forms, and disruption in protein folding and trafficking.^55^ However, excessive glucose levels may also lead to dysregulation of glycosylation patterns, contributing to aberrant glycoprotein processing and diseases.^56^ Mannose is a core component of the glycan structure and can bypass glucose to support high-mannose glycosylation^57^; it can rescue glycosylation defects under glucose-limited conditions. Fructose can be metabolized into intermediates like mannose-6-phosphate, modestly supporting glycan synthesis, though less efficiently than glucose. Pentoses, such as ribose, contribute minimally and indirectly through the pentose phosphate pathway but are not sufficient to maintain functional N-glycosylation. Together, these sugars influence glycosylation outcomes based on their metabolic integration and availability.^55^

In the present study, we demonstrated that the kinetic heterogeneity in the inactivation delay of Kv3.4 is due to its N-glycosylation state. We observed that in heterologous systems, Kv3.4 is expressed in different N-glycosylated forms, with each N-glycoform presenting a specific inactivation delay. Consequently, we found that variation in glucose availability strongly affects Kv3.4 N-glycosylation and, in consequence, its inactivation kinetics. Finally, we showed that in the absence of glucose, supplementing with glucose or mannose restores the N-glycosylation and range of inactivation delay of Kv3.4, whereas supplementing with pyruvate or ketone bodies (KBs), the main alternative fuel suppliers of the brain,^58^ does not.

We also showed that N-glycosylation affects not only Kv3.4 but also other Kv channels that have predicted N-glycosylation sites and show variability in inactivation kinetics. In contrast, Kv channels that lack N-glycosylation sites do not exhibit this glycosylation-dependent variability in their inactivation kinetics. These findings support the notion that N-glycosylation acts as an additional mechanism for modulating the kinetics of ion channels. Furthermore, since N-glycosylation is closely linked to glucose availability, our results suggest that fluctuations in glucose levels could directly impact Kv channel behavior, thereby influencing neuronal excitability and signaling dynamics in the nervous system.

## Results

### N-glycosylation sites and heterogeneity in inactivation delay of rat Kv3.4

**Figure 1:**
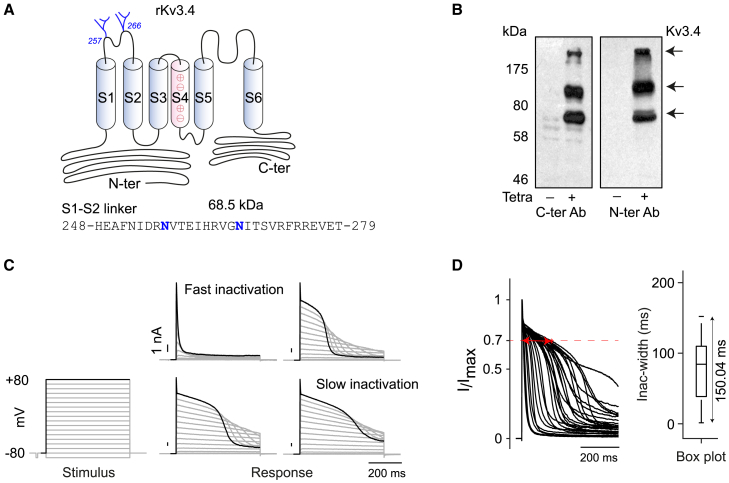
N-glycosylation sites and heterogeneity in inactivation delay of rat Kv3.4 (A) Schematic representation of the rat Kv3.4 (rKv3.4) channel, showing two predicted N-glycosylation sites (blue) in the S1-S2 extracellular loop. The amino acid sequence of this region, including both N-glycosylation motif, is shown below. (B) Western blot of CHO rKv3.4 cells with (+) or without (−) tetracycline induction, probed with N- and C-terminal Kv3.4 antibodies, revealing bands at ∼70 kDa, ∼100 kDa and >200 kDa. (C) Representative current traces from four different CHO rKv3.4 cells, showing a range of inactivation delay in response to an 18-step depolarization protocol (−90 mV to +80 mV, 500 ms, 25°C). (D) Overlay of evoked current traces at +80 mV (*n* = 29), spanning 70 ms–599 ms and normalized to each cell’s maximum current. Boxplot analysis highlights the variability in inactivation delay using the inactivation width (red arrow). The whisker range (150.04 ms) is used as a quantitative measure of inactivation delay heterogeneity.

The S1-S2 loop region of all Kv3 members contains two N-glycosylation sites.^45^ For rat Kv3.4 (rKv3.4), these sites are located at N257 and N266 (Figure 1A). Western blot analysis of CHO cells expressing rKv3.4 with or without tetracycline induction confirmed channel expression and revealed three prominent bands ranging from ∼70 to >200 kDa (Figure 1B). The lower two bands likely represent distinct glycoforms of Kv3.4, while the uppermost band may correspond to Kv3.4 tetramer or a Kv3.4 complex associated with auxiliary subunits. Electrophysiological recordings from CHO cells overexpressing rKv3.4, showed a wide range of inactivation delay kinetics, spanning from fast to slow inactivation in response to the very same stimulus (Figure 1C). The heterogeneity is visually depicted through an overlay plot that displays normalized currents (I/I_max_) in response to a +80 mV voltage stimulus. Here, “heterogeneity” refers to the absolute spread of the data, which encompasses effects on both the mean inactivation width and the variation in inactivation width. To quantify this heterogeneity, first the inactivation width (Inac-width) is measured at 70% of the current amplitude (Figure 1D). Inact-widths from multiple cells (*n* > 20) are then represented using a boxplot, where the distance between the whiskers is used as a metric for heterogeneity. A broader whisker span indicates greater heterogeneity, while a narrower one suggests less. This heterogeneity in inactivation for Kv3.4 in CHO is conserved at different temperatures (15°C and 35°C, Figure S2A), across species (human and mouse, Figure S2B), and is not influenced by the patch-clamp methods (Figure S2C). Furthermore, time-course experiments varying tetracycline induction from 4 h to 72 h (Figures S2D and S2E), along with correlation analysis between Inactivation width and I_max_ (Figure S2F), support that the observed kinetic heterogeneity is an inherent feature of the Kv3.4 channel. However, the reason behind this heterogeneity has not yet been reported or investigated.

### N-glycosylation modulates the inactivation delay kinetics of Kv3.4

**Figure 2:**
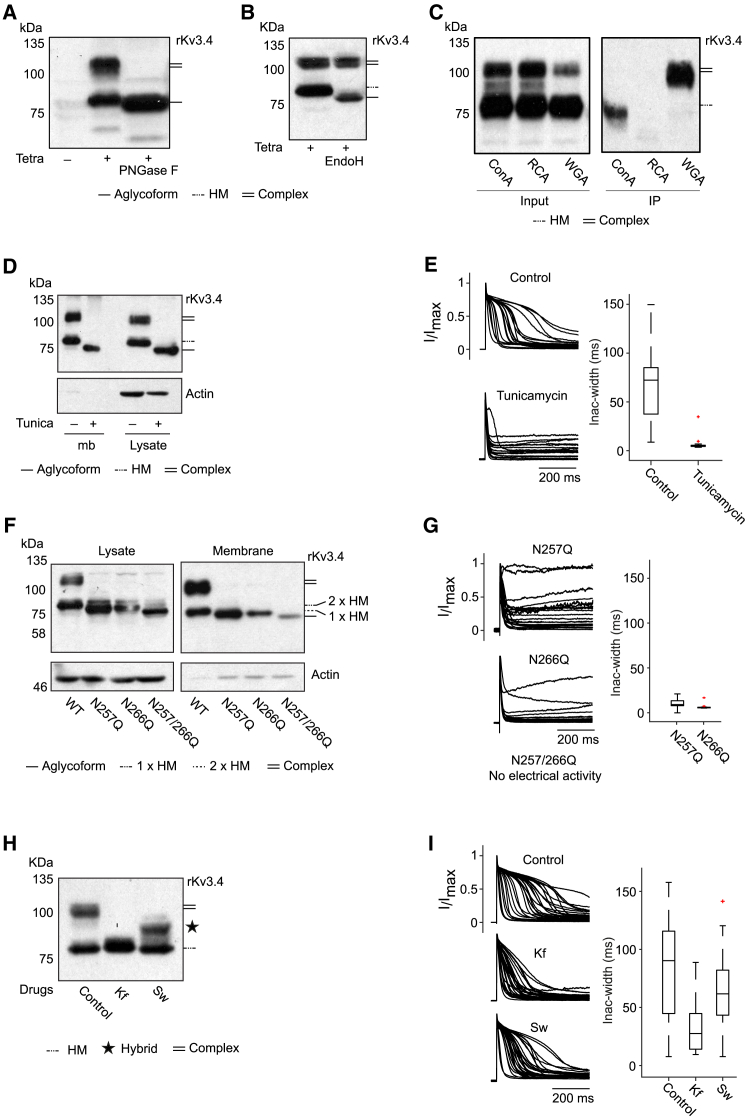
N-glycosylation modulates the inactivation delay kinetics of Kv3.4 (A and B) Western blot analysis of cell lysates from CHO rKv3.4 cells induced with tetracycline and treated with PNGase F (A) or EndoH (B), showing changes in glycoform profiles. (C) Lectin-binding assays using ConA, RCA, and WGA on CHO rKv3.4 cells, detecting high-mannose, and complex glycans, respectively. Immunoprecipitated samples (IP) and total input controls were analyzed by western blot. (D) Surface biotinylation of CHO rKv3.4 cells treated or untreated with tunicamycin, followed by western blot analysis of membrane (mb) and total lysate fractions, demonstrating that tunicamycin fully blocks Kv3.4 glycosylation. (E) Impact of tunicamycin treatment on CHO rKv3.4 inactivation delay, shown by overlay plots and boxplot analysis (control, *n* = 20; tunicamycin, *n* = 19). (F) Western blot analysis of total lysate and membrane proteins from cell surface biotinylation of CHO cells expressing rKv3.4 N-glycosylation site mutants (N257Q, N266Q, N257/266Q). (G) Overlay plots of the rKv3.4 mutants (N257Q, *n* = 23; N266Q, *n* = 16; N257/266Q, *n* = 34) and boxplot analysis. (H) Western blot analysis of CHO rKv3.4 cells untreated (control) or treated with kifunensine (Kf) or swainsonine (Sw), indicating the presence of different glycoforms. Star indicate putative hybrid glycoform. (I) Effects of Kf and Sw treatments on rKv3.4 inactivation delay, shown with overlay plots and quantified by boxplots (control, *n* = 29; Kf, *n* = 29; Sw, *n* = 29). In F, “1× HM” and “2× HM” refer to the number of N-glycosylation sites occupied by high-mannose glycans. Western blotting was performed using an anti-Kv3.4 C-terminal antibody.

To verify that the higher ∼100 kDa band corresponds to the glycosylated form of rKv3.4, we performed enzymatic tests using peptide-N-glycosidase F (PNGase F) and endoglycosidase H (EndoH), which respectively removes all types of N-glycans or only oligo-mannose and some hybrid glycans. As shown in Figure 2A, both ∼75 kDa and ∼100 kDa bands are sensitive to PNGase F treatment, leading to a single lower band, closer to the predicted size (68.5 kDa) of rKv3.4, suggesting that both bands contain N-glycans. However, only the ∼75 kDa band is sensitive to EndoH (Figure 2B), indicating that this latter contains oligo-mannose and possibly some hybrid types. Further lectin binding assay confirmed the composition of the different bands. Lectins are glycan-binding proteins (GBPs) that selectively recognize glycan epitopes of glycoproteins (concanavalin A is specific for oligo-mannose, Ricinus communis agglutinin: RCA is specific for hybrid types, and wheat germ agglutin: WGA is specific for complex glycans). As shown in Figure 2C, the lower band is precipitated with concanavalin A, whereas the higher band is precipitated with WGA, suggesting that the lower band contains oligo-mannose and the higher band contains complex glycans, confirming the previous observations. No signal was detected after incubation with RCA, suggesting that the expression of hybrid-linked types is not significant.

Then to investigate the effect of N-glycosylation on Kv3.4 kinetics, we treated the CHO rKv3.4 with tunicamycin, a strong inhibitor of N-glycosylation which prevents the transfer of the N-glycan precursor to protein during its synthesis in the ER (Figure S1).^59^ As expected, tunicamycin blocks all types of glycosylation, resulting in the exclusive expression of the non-glycosylated form of rKv3.4 (aglycoform) (Figure 2D) without affecting its translocation to the membrane. The overlay plot of normalized currents and the range of inactivation widths (boxplot) show that tunicamycin turns rKv3.4 into a fast-inactivating channel and suppresses the heterogeneity in the inactivation delay (Figure 2E).

To confirm that the loss of heterogeneity in rKv3.4 inactivation delay is directly linked to the inhibition of rKv3.4 glycosylation and not from an off-target effect of tunicamycin, we generated the mutant versions of rKv3.4 N-glycosylation sites (N257Q, N266Q, and N257/266Q), and assessed their electrical behaviors. Protein analysis shows that the mutation of a single glycosylation site is sufficient to completely inhibit the complex glycosylation at both sites (N257Q, N266Q; Figure 2F left panel), and the mutations of both N-sites lead, as expected, to the exclusive production of the aglycoform (N257/266Q; Figure 2F). Similarly, these mutations have little impact on the localization of the ion channel at the membrane (Figure 2F, right panel). The overlay plot of voltage-clamp recordings and the boxplot of inactivation width of both single mutants shows the suppression of the heterogeneity in the inactivation delay kinetics (Figure 2G), similar to that observed in the presence of tunicamycin (Figure 2E). Interestingly, the double mutant N257/266Q becomes electrically silent. It is possible that not only the inhibition of N-glycosylation but also the introduced mutations (two N converted to Q) are responsible for the suppression of Kv3.4 electrical activity.

To further examine the inactivation kinetics of the different glycoforms of rKv3.4, we used drugs known to affect specific enzymatic steps of the N-glycosylation process, such as kifunensine (Kf) and swainsonine (Sw). Kf inhibits the formation of hybrid and complex glycans, whereas Sw impairs the formation of complex glycans (Figure S1).^60^ As expected, treatment with Kf impairs the formation of complex glycoforms with preservation of the high-mannose (HM) glycoforms, whereas treatment with Sw impairs the formation of complex glycoforms with the appearance of additional intermediates, most probably some hybrid types (Figure 2H). The overlay plot of normalized currents and the range of inactivation widths (boxplot) reveals that the cells treated with Kf show reduced heterogeneity in the inactivation delay in comparison with the control cells (Figure 2I, Kf), reflecting the kinetics of the HM glycoform (or core-glycosylated form) of rKv3.4. Both the overlay plot and the box-plot corresponding to the Kv3.4 expressing cells treated with Sw show a pattern in between the non-treated cells and Kf-treated cells, (Figure 2I, Sw). These observations suggest that the different glycoforms of Kv3.4 exhibit their own kinetic behavior and that the N-glycosylation status of Kv3.4 is responsible for the range of inactivation delay kinetics. To confirm the specific role of N-glycosylation in modulating Kv3.4 kinetics, we tested the effect of thapsigargin, an ER stress inducer also known to interfere with the glycosylation process,^61^ alongside two control compounds unrelated to N-glycosylation: the antioxidant N-acetylcysteine (NAC) and the mTOR inhibitor rapamycin. Neither NAC nor rapamycin altered Kv3.4 glycosylation or its inactivation delay kinetics (see NAC and Rapa in Figure S3), whereas thapsigargin affected both (see Thapsi in Figure S3), confirming that Kv3.4 N-glycosylation and heterogeneity in the inactivation delay are closely interconnected.

To summarize, we discovered that rKv3.4 is expressed in different N-glycoforms when overexpressed in CHO cells, and that its N-glycosylation status is directly linked to its range of inactivation delay kinetics. We used mutants or specific drugs to restrict the expression of Kv3.4 to its aglycoform or high-mannose form (or core-glycosylated form), and found that the aglycoform presented the fastest and most stable inactivating pattern, and the HM form, a relatively fast and consistent inactivation pattern. Unfortunately, it was not possible to experimentally constrain the expression of Kv3.4 to its complex glycoforms. However, through comparison with the other forms, we are able to deduce that complex Kv3.4 glycoforms behave as slowly inactivating channels, with a range of inactivation delays that may depend on the type of complex glycans attached. Indeed, multiple bands were observed ∼100 kDa, suggesting that more than one type of complex N-glycans is attached to Kv3.4, in accordance with the notion of glycosylation microheterogeneity or the fact that a particular N-glycosylation site may be occupied by several structurally distinct glycans.^62^

### Glucose availability modulates N-glycosylation and inactivation delay kinetics of rat Kv3.4

Glucose and fructose serve as primary precursors for the synthesis of monosaccharides, which form the backbone of glycan trees.^41^ Hence, a change in glucose concentration should affect the production of glycans, the glycosylation of Kv3.4 and consequently, its inactivation kinetics.

**Figure 3:**
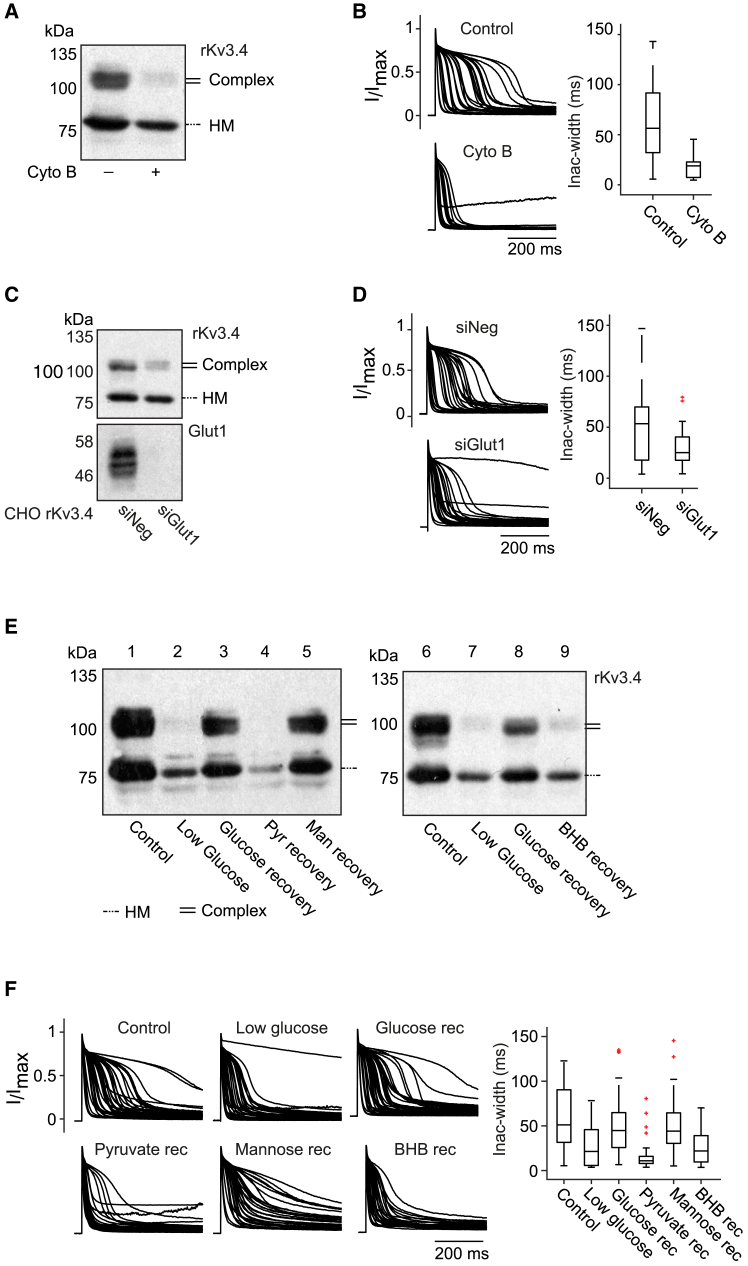
Glucose availability modulates the N-glycosylation and the inactivation delay kinetics of rat Kv3.4 (A) CHO rKv3.4 cells were treated with (+) or without (−) cytochalasin B (Cyto B), an inhibitor of glucose transport. Western blot analysis shows that glucose uptake inhibition impairs rKv3.4 complex glycosylation. (B) The effect of Cyto B on rKv3.4 inactivation heterogeneity is shown with overlay plots and quantified using boxplots (control, *n* = 29; Cyto B, *n* = 19). (C) CHO rKv3.4 cells were transiently transfected with siRNA targeting GLUT1 (siGlut1) or a non-targeting control (siNeg). Western blot confirms the absence of GLUT1 protein expression and its effect on Kv3.4 glycosylation. (D) Heterogeneity in inactivation delay in both cases (siNeg and siGlut1) are shown with overlay plots and quantified using boxplots (siNeg, *n* = 26; siGlut1, *n* = 25). (E) CHO cells expressing rKv3.4 were cultured in normal or low-glucose media, or in low-glucose media followed with subsequent recovery with different compounds (glucose, pyruvate, mannose, or β-hydroxyburyrate:BHB). Western blot analysis shows how different recovery conditions affect glycosylation profiles. (F) Heterogeneity in inactivation delay of rKv3.4 under each condition are shown with overlay plots and quantified using boxplots (control, *n* = 41; low glucose, *n* = 39; glucose recovery, *n* = 41; pyruvate recovery, *n* = 27; mannose recovery, *n* = 35; BHB recovery, *n* = 23).

We tested this hypothesis using cytochalasin B (cyto B), a non-selective inhibitor of glucose transporter (GLUT). Our results showed that cyto B impaired the complex glycosylation of Kv3.4 but not the production of HM glycoforms (Figure 3A). It is possible that other sources of sugars (e.g., fructose, mannose, and galactose), whose transport is not affected by cyto B, are present in the culture medium and sufficient for the synthesis of HM types glycans. The overlay plot and the box-plot show that the kinetics of Kv3.4 is strongly affected by cyto B (Figure 3B), leading to a faster inactivating channel, close to what was observed with the use of kifunensine (Figure 2I), that also restricted the expression of Kv3.4 to its HM-glycoform.

Glucose transporter 1 (GLUT1) is the key transporter of glucose from blood to brain cells.^63^ As shown by transcriptomic analysis, GLUT1 is the member of the GLUT family with the highest expression in CHO cells (Table S2). Accordingly, we wonder whether N-glycosylation and kinetics of Kv3.4 could be affected by specific GLUT1 deficiency. We used siRNA to silence the *Slc2a1* gene in CHO cells, which efficiently abolishes the production of the GLUT1 protein (Figure 3C, bottom panel). We observed that specific deficiency in GLUT1 strongly impairs the complex glycosylation of Kv3.4 (Figure 3C, upper panel) as well as the kinetic properties of Kv3.4, that presents with faster inactivation and reduced kinetic heterogeneity (Figure 3D).

Then, to mimic a more physiologically relevant condition, we cultured CHO rKv3.4 cells in low-glucose medium prior to channel induction. As shown in Figure 3E (lanes 1–2), glucose deprivation led to a strong reduction in complex glycoforms of Kv3.4, while HM glycoforms band remained visible. Functionally, glucose deprived cells exhibited faster inactivation and reduced kinetic heterogeneity, as illustrated by the overlay and boxplots (Figure 3F).

To determine whether this effect is reversible, we reintroduced glucose at the time of Kv3.4 expression. This restored both complex glycosylation (Figure 3E, lane 3) and the heterogeneous inactivation phenotype (Figure 3F), confirming that glucose deprivation affects Kv3.4 glycosylation and function in a reversible manner. In contrast, supplementation after glucose deprivation with pyruvate or β-hydroxybutyrate (BHB), two alternative sources of fuel for the brain in case of glucose deprivation,^64^^,^^65^ did not rescue complex glycosylation (Figure 3E, lanes 4, 9) or heterogeneity in inactivation (corresponding plots in Figure 3F). This is in agreement with the fact that neither pyruvate nor BHB is a potent precursor for glycans or glucose synthesis. Interestingly, supplementing with mannose, a critical precursor for N-glycan synthesis,^57^ successfully restored both complex glycoforms and the inactivation heterogeneity.

Additionally, a higher concentration of glucose does not significantly affect the glycosylation or the kinetic heterogeneity of Kv3.4 (Figures S4A and S4B). This effect of glucose on Kv3.4 kinetic modulation is conserved across species, as mouse and human Kv3.4 also show a glycosylation defect, faster inactivation, and significantly reduced heterogeneity in inactivation delay upon tunicamycin treatment and glucose deprivation (Figures S4C–S4F).

### Kv3.4 is only expressed in complex glycoforms in the rodent brain

**Figure 4:**
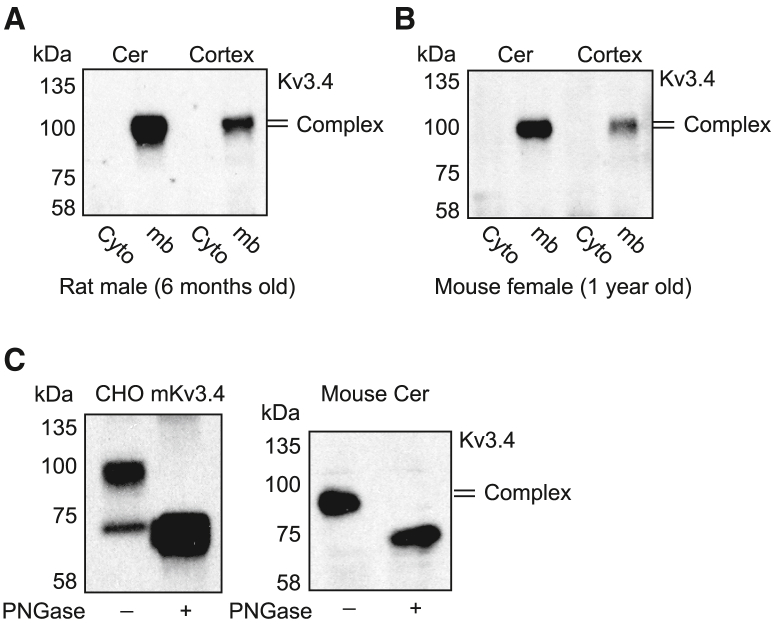
Kv3.4 glycosylation pattern in rodent brain tissue (A and B) Western blot analysis of Kv3.4 glycosylation in brain tissues from (A) 6-month-old male rats and (B) 1-year-old female mice. Membrane (mb) and cytoplasm (cyto) were isolated from cerebellum (Cer) and cortex tissues, followed by Kv3.4 immunoprecipitation using N-terminal Kv3.4 antibody and blotting with a C-terminal Kv3.4 antibody. Complex glycosylated Kv3.4 bands were only detected in membrane extracts. (C) Western blot analysis of isolated membrane fractions from CHO cells expressing mouse Kv3.4 and from mouse cerebellum tissue. Samples were immunoprecipitated with an N-terminal Kv3.4 antibody and treated with (+) or without (−) PNGase F. Blotting was performed using C-terminal Kv3.4 antibody. PNGase F treatment confirmed the presence of complex glycosylated Kv3.4 in brain tissue.

We have shown that Kv3.4 is expressed in different N-glycoforms upon overexpression in a heterologous system, each of them presenting a different kinetic profile in inactivation. Hence, we investigated which glycoforms of Kv3.4 is expressed in the rodent brain tissue. To this aim, we first purified membranes from brain tissues and immunoprecipitated Kv3.4 before detection by western blot (Figure S5). A unique band ∼100 kDa, corresponding to the complex glycoform of Kv3.4, was detected, irrespective of age, brain regions (cortex or cerebellum), gender or species (Figures 4A and 4B). HM glycoform was completely absent on isolated membranes of the brain tissue. PNGase F treatment after immunoprecipitation further confirmed that the immunoprecipitated band corresponds to the glycosylated form of Kv3.4 (Figure 4C). These results indicate that only complex N-glycoforms of Kv3.4 are expressed at the membrane of the rodent brain. Based on our analyses in CHO cells, we can predict that in physiological conditions, Kv3.4 would exhibit slow-inactivating kinetic in brain cells.

### N-glycosylation explains kinetic heterogeneity in other Kv channels

**Figure 5:**
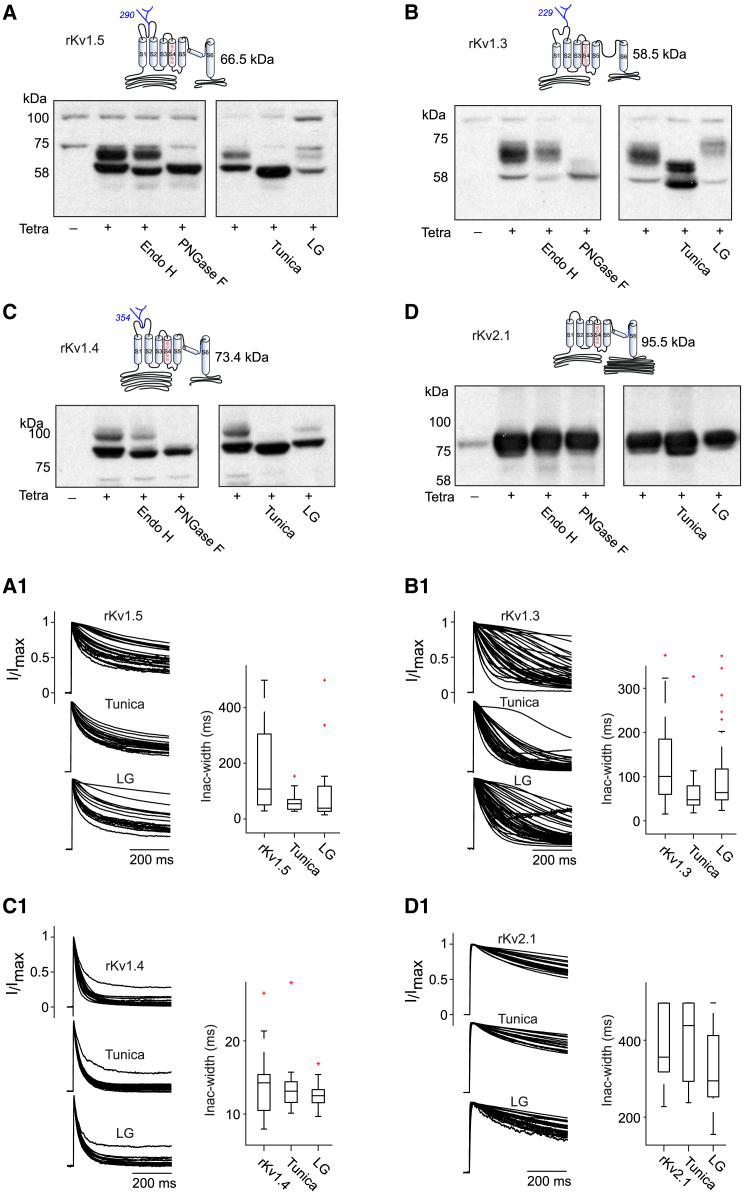
N-glycosylation explains kinetic heterogeneity for other Kv channels (A–D) Schematic diagrams indicate the predicted N-glycosylation sites (in blue) in rat Kv1.5 (A), Kv1.3 (B), Kv1.4 (C), and Kv2.1 (D); Kv2.1 lacks predicted glycosylation sites. Western blot analyses of CHO cells expressing each channel after treatment with PNGase F, EndoH, tunicamycin, or culture in low-glucose (LG) medium reveal distinct glycoform patterns and varying sensitivity to glycosylation disruption. No effect is observed for Kv2.1. (A1–D1) Overlay and boxplots of inactivation width demonstrate that glycosylation disruption (via tunicamycin or LG treatment) reduces kinetic heterogeneity in Kv1.5, Kv1.3, and Kv1.4 (*n* = 29,19,19; 43, 36, 43; 21, 22, 18 for control/Tunica/LG, respectively). Kv2.1 shows no change in glycosylation pattern or kinetics (*n* = 19, 18, 30 for control/Tunica/LG, respectively), consistent with the absence of glycosylation sites.

We have shown that N-glycosylation plays a crucial role in regulation of Kv3.4 kinetics. Interestingly, we have found that other Kvs, such as Kv1.5 and Kv1.3 which also exhibit high heterogeneity in kinetics^21^ hold two N-glycosylated sites on their S1-S2 loop, similar to Kv3.4. To determine if N-glycosylation is a general mechanism of kinetic modulation, we tested the effect of N-glycosylation on the kinetics of these other heterogeneous channels. Our findings show that tunicamycin treatment and glucose deprivation affect both the N-glycosylation and the kinetics of these channels, resulting in reduced heterogeneity and faster inactivation (Figures 5A, 5B, 5A1, and 5B1), such as observed for Kv3.4. We also tested the effect of N-glycosylation on the fast-inactivating Kv1.4 channel that does not present heterogeneity, and found that the kinetics of Kv1.4 becomes even more stable when glycosylation is impaired without affecting its overall kinetic properties (Figures 5C and 5C1). Finally, we tested a non-glycosylated channel (Kv2.1) as a control, and found that its kinetics is not affected by tunicamycin nor glucose deprivation (Figures 5D and 5D1), demonstrating that the effect observed for glycosylated channels is specific to N-glycosylation disturbance.

In conclusion, our research suggests that N-glycosylation is responsible for the modulation of kinetics in specific Kv channels, explaining the observed kinetic heterogeneity upon overexpression in CHO cells.^21^

## Discussion

In our previous study,^21^ we observed significant heterogeneity in the inactivation delay kinetics of Kv3.4 channel. In this study, we found that N-glycosylation is responsible for this heterogeneity in kinetics of Kv3.4, without affecting its membrane trafficking. We have also found that the availability of glucose has a strong modulatory effect on Kv3.4 kinetics. Importantly, we show that this modulatory effect of glucose on Kv3.4 inactivation kinetics is reversible. Moreover, we have observed that, not only Kv3.4 but other Kv channels such as Kv1.5 and Kv1.3 are modified by N-glycosylation, and that their kinetics is also sensitive to glucose concentration. These findings suggest that changes in N-glycosylation can dynamically influence the kinetic of Kv3.4 and other Kv channels, ultimately affecting the overall electrical behavior of excitable cells.

We demonstrate that, in CHO cells, the high-mannose form of Kv3.4 exhibits consistent fast inactivation, whereas the complex glycoforms display a range of slow-inactivating patterns. Our experimental data suggest that various types of complex glycans are present on the surface of the protein, which may define the speed of inactivation of the channel. Indeed, western blot analyses show several bands ∼100 kDa indicating that more than one type of complex N-glycoforms are present. In addition, an intermediate form of glycosylation correlates with an intermediate pattern of inactivation (Figures 2H and 2I; Sw treatment). Finally, Kv3.4 overexpressed in other cell lines (HEK and CV1) presents distinct patterns of complex N-glycosylation that correlates with distinct ranges of inactivation (Figure S6). Since the possible number of different complex glycans is immense, (see Figure S1;^41^), they could hence confer an enormous diversity, even if subtle, in the kinetics of Kv3.4.

Heterologous expression systems have been instrumental in studying ion channel glycosylation and its effect on surface expression and function. CHO cells, in particular, are known for producing a full spectrum of glycoforms.^52^^,^^66^ For Kv3 channels neuroblastoma (NB) and Spodoptera frugiperda (Sf9) cell lines have been used to show that N-glycosylation directly influences both the subcellular distribution and the biophysical properties of Kv3 channels.^48^^,^^49^ We acknowledge that our use of CHO cells, introduces differences compared to physiological conditions. While CHO cells are capable of producing complex-glycosylated proteins, they also allow the expression of high-mannose and hybrid glycoforms, which are not typically observed in brain tissue. However, the coexistence of multiple glycoforms in the CHO cells enabled us to systematically dissect how glycosylation profiles influence inactivation kinetics; an effect that would be difficult to observe in native tissue, where only complex glycoform is present. Moreover, it is important to note that studying a single ion channel, such as Kv3.4, in native cells is experimentally challenging, as multiple VGICs are co-expressed simultaneously. Therefore, a controlled expression system like CHO cells remains a reliable model to isolate and characterize channel kinetics and to investigate the effect of glycosylation on a single ion channel.

N-glycosylation is a complex process that involves a multitude of enzymes expressed depending on various factors, including tissues, cell types, and development stage, among others. It is the combination of these specific enzymes present in the cell that determines the types of glycans attached to proteins in this specific cell. It is therefore likely that the diversity in the glycan composition is an additional mechanism to fine-tune the kinetics of those ion channels. The role of N-glycosylation on cell surface expression has been controversial. While some studies^67^ support a clear role for glycosylation in promoting membrane trafficking, others^48^^,^^49^ report no significant effect on surface expression. Our data show that mutating a single N-glycosylation site does not impair Kv3.4 membrane localization or function as evidenced by measurable whole-cell currents. However, when both glycosylation sites are mutated (N257Q/N266Q), the protein is still present at the membrane, but the channel becomes electrically silent. We hypothesize that this double mutation may cause misfolding, disrupting channel function despite successful trafficking to the membrane. In contrast, single-site mutants likely preserve enough structural integrity to maintain function. Interestingly, we also observed that current density in the single mutants is significantly reduced compared to wild-type Kv3.4. This reduction suggests that while glycosylation may not be strictly required for surface expression, it likely enhances membrane stability or trafficking efficiency, ultimately influencing Kv3.4 functional expression levels.

From our investigations on the N-glycosylation pattern of endogenous Kv3.4 in the rodent brain, we found that Kv3.4 is modified with complex N-glycans only, unlike in heterologous systems. This suggests that in brain cells, Kv3.4 behaves as a slow-inactivating channel, with a range of inactivation that may depend on the type of complex glycans attached to the protein. The exact mechanism by which different glycosylation patterns cause these biophysical changes is still unknown. However, it is likely that the types of glycans attached to the surface of the channel affect its conformation, similar to how phosphorylation of its NTID domain affects N-type inactivation.^31^ Further studies are necessary to establish this mechanism of action.

In the present study, we have focused on Kv3.4 because of its remarkable heterogeneity in inactivation kinetics, but we also showed that a disturbance in N-glycosylation affects the kinetics of other Kv channels. Hence, N-glycosylation may be a universal mechanism for modulating ion channel kinetics, as other types of ion channels, such as voltage-gated calcium (Cav) or voltage-dated sodium (Nav) channels that are also known to be glycosylated and affected by changes in glucose concentrations.^47^^,^^68^^,^^69^^,^^70^

Our findings suggest that variations in glucose concentrations can directly affect the kinetic properties of Kv3.4 and we have some indications that other glycosylated channels could also be affected. We predict that, in pathological conditions, disturbances in glucose metabolism could affect the kinetics of any ion channel modified by N-glycosylation, which may contribute to neurological symptoms observed in such conditions or diseases. This prediction is supported by the fact that neurological defects (mental retardation, seizure, epilepsy, microcephaly, and ataxia) are frequently observed in patients with congenital disorders of glycosylation (CDGs), rare conditions due to mutations in genes encoding key components of the glycosylation machinery.^71^

Glucose is the main source of energy for the brain and its concentration must be tightly regulated.^72^ Several studies have demonstrated the link between disturbances in glucose metabolism and various neurological and psychiatric disorders. Sporadic hypoglycaemia may provoke poor attention and cognitive dysfunction, while excessive glucose consumption may contribute to memory deficits.^73^^,^^74^ Hypo and hyperglycemia due to dysregulation in glucose metabolism, as for example in diabetes mellitus, is thought to damage neurons.^75^ GLUT1 deficiency syndrome (GLUT1 DS), a rare genetic condition caused by impaired glucose transport into the brain mediated by GLUT1, the glucose transporter at the blood-brain barrier, is associated with early-onset epilepsy, developmental delay, complex movement disorders, and sometimes microcephaly.^76^^,^^77^ More recently, hypometabolism (decreased glucose consumption) was also recognized as the primary initiating factor for many neurodegenerative diseases, such as Alzheimer’s disease, Parkinson’s disease, or acquired epilepsy.^78^ While it is commonly assumed that neurological damage associated with a disturbance in glucose availability or metabolism, is mainly caused by a deficit in ATP production in the brain,^79^ our study suggests that alteration in glycosylation of ion channels and consequently ion channels kinetics must also be considered as a potential mechanism of action.

As exemplar, the gold standard therapy for GLUT1 DS patients is the ketogenic diet^80^^,^^81^; an intervention that induces the production of KBs by the body, used as an alternative source of energy for ATP production. In GLUT1 DS patients, dietary induction of ketogenesis is effective in reducing seizures, but the effects on neurodevelopment or movement disorders are less impressive.^82^ KBs are fueling the Krebs cycle to allow ATP synthesis in mitochondria, but are biochemically unable to compensate for a glycosylation deficit, as we confirmed in the present study. It is possible that, *in vivo*, the use of KBs for oxidative metabolism “frees up” glucose for other important functions such as cytoplasmic antioxidant defense system as proposed by Stincone et al.,^83^ or for glycan synthesis and glycosylation.^84^

In conclusion, our study demonstrates that N-glycosylation is a dynamic modulator of ion channel kinetics, highlighting the emerging notion that N-glycosylation plays a crucial role in regulating the electrical activity of cells.^85^^,^^86^ In consequence, N-glycosylation needs to be considered as a potential contributing mechanism in the neurological disorders associated with glucose metabolism disturbances.

### Limitations of the study

Our study provides strong evidence that N-glycosylation modulates the inactivation kinetics of Kv3.4 and other glycosylated potassium (Kv) channels. However, two main limitations should be noted. First, all experiments were conducted in a heterologous expression system (CHO cells). While this approach is useful for mechanistic dissection, it does not fully replicate the native glycosylation environment found in neurons. As a result, the direct application of our findings to physiological or pathological conditions in the brain may be limited. Second, although we established a clear correlation between the N-glycosylation status and the inactivation delay, the specific structural and biophysical mechanisms through which individual glycans influence Kv3.4 gating remain unclear. While the observed Kv3.4 current waveforms can be quantitatively modeled using potassium (K^+^) dynamics and Hodgkin-Huxley modeling, the study does not provide a mechanistic explanation for the current shapes recorded from Kv3.4-expressing cells. Further structural studies and single-channel analyses are necessary to clarify how different glycan structures modulate channel behavior at the molecular level.

## Resource availability

### Lead contact

Further information and requests for resources should be directed to and will be fulfilled by the lead contacts, Dr. Rajnish Ranjan (rajnish.ranjan@gmail.com).

### Materials availability

This study did not generate new unique reagents.

### Data and code availability

The raw electrophysiological recordings and western blot image files generated in this study have been deposited in the Mendeley Data repository and are publicly available at https://doi.org/10.17632/vwnzy3trrr.2 Electrophysiology datasets and analysis code will be made available via Channelpedia (https://www.channelpedia.net/) upon publication. Any additional data or materials required to reanalyze the findings of this study are available from the lead contact upon reasonable request.

## Acknowledgments

We thank Magali Joffraud, Lionel Ponsonnet, and Manuel Hernandez for their valuable experimental support, and Charlotte Lorin for performing manual patch-clamp recordings. We are also grateful to the neighboring laboratory of Prof. Ralf Schneggenburger for kindly providing C57BL/6J mice, which enabled us to assess the glycosylation profile of Kv3.4 in rodent brain tissue.

This study was supported by funding to the Blue Brain Project, a research center of the École polytechnique fédérale de Lausanne (EPFL), from the Swiss government’s ETH Board of the Swiss Federal Institutes of Technology.

## Author contributions

R.R.: conceptualization, data curation, formal analysis, investigation, methodology, project administration, supervision, validation, visualization, writing – original draft, writing – review and editing; E.L.: conceptualization, data curation, investigation, methodology, project administration, supervision, validation, writing – original draft; M.H.: investigation; V.B.: investigation; E.S.: software, data curation; H.M.: conceptualization, funding acquisition, supervision, writing – original draft.

## Declaration of interests

The authors declare no competing interests.

## STAR★Methods

### Key resources table

**Table:** 

REAGENT or RESOURCE	SOURCE	IDENTIFIER
**Antibodies**		

anti-Kv3.4-C-terminus	LSBio	Cat# LS-C165415
anti-Kv3.4-N-terminus	UC Davis/NIH NeuroMab Facility	Cat# N72/16; RRID:AB_2877318
anti-Kv3.3	LSBio (LifeSpan)	Cat# LS-C101533-50; RRID:AB_10569184
anti-Glut1	Abcam	Cat#ab15309; RRID:AB_301844
anti-Kv1.3	UC Davis/NIH NeuroMab Facility	Cat# L23/27; RRID:AB_2877316
anti-Kv1.5	Abcam	Cat# ab110469; RRID:AB_10866275
anti-Actin	Abcam	Cat# ab3280; RRID:AB_303668
Goat anti-mouse-HRP	Jackson ImmunoResearch	RRID:AB_10015289
Goat anti-rabbit-HRP	Biorad	1662408EDU

**Chemicals, peptides, and recombinant proteins**		

Biotinylated Concanavalin A	Vector Laboratories	Cat# B-1005
Biotinylated Ricinus Communis Agglutinin I	Vector Laboratories	Cat# B-1085
Biotinylated Wheat germ agglutinin	Vector Laboratories	Cat# B-1025
Peptide N-Glycosidase F (PNGase F)	New England BioLabs	Cat# P0704L
Endoglycosidase H (Endo H)	New England BioLabs	Cat# P0702S
Tetracycline (Doxycycline hyclate)	Sigma-Aldrich	Cat# D9891-5G
Tunicamycin	Sigma-Aldrich	Cat# T7765
Swainsonine	Tocris	Cat# 3208
Kifunensine	Tocris	Cat# 3207
Rapamycin	Enzo Life Sciences	Cat# BML-A275
N-acetyl-L-cysteine (NAC)	Sigma-Aldrich	Cat# A7250
Thapsigargin	Enzo Life Sciences	Cat# BML-PE180
Cytochalasin B	Sigma-Aldrich	Cat# C2743
D(+)Mannose	Sigma-Aldrich	Cat# M6020
Sodium Pyruvate	Thermo Fisher Scientific	Cat# 11360070
3-hydroxybutyric acid (BHB)	Sigma-Aldrich	Cat# 166898
RPMI 1640	Gibco	Cat# 21875034
RPMI no glucose	Gibco	Cat# 11879020
RPMI 1640 high glucose	Gibco	Cat# A1049101
Fetal calf serum	Gibco	Cat# 10270106
NaCl	Sigma-Aldrich	Cas# 7647-14-5
KCl	Sigma-Aldrich	Cas# 7447-40-7
MgCl2	Sigma-Aldrich	Cas# 7791-18-6
CaCl2	Sigma-Aldrich	Cas# 10035-04-8
D-Glucose	Sigma-Aldrich	Cas# 50-99-7
HEPES (free acid)	Sigma-Aldrich	Cas# 7365-45-9
KF	Sigma-Aldrich	Cas# 7789-23-3
EGTA (free acid)	Sigma-Aldrich	Cas# 67-42-5

**Critical commercial assays**		

jetPRIME® Transfection Reagent	Polyplus	Cat# 114-15
GeneArt™ Site-Directed Mutagenesis System	Thermo Fisher Scientific	Cat# A13282
Neon® transfection system, 100 μL	Thermo Fisher Scientific	Cat # MPK10025
Mem-Per™ Plus Membrane Protein Extraction Kit	Thermo Fisher Scientific	Cat # 89842
Pierce™ Cell Surface Protein Isolation Kit	Thermo Fisher Scientific	Cat# 89881
Pierce 660 nm Protein assay Reagent	Thermo Fisher Scientific	Cat# 22662
Pierce™ ECL Western Blotting Substrate	Thermo Fisher Scientific	Cat# 32106

**Deposited data**		

Electrophysiology & WB	Mendeley Data	https://doi.org/10.17632/vwnzy3trrr.2

**Experimental models: Cell lines**		

Flp-In™ CHO	Thermo Fisher Scientific	RRID: CVCL_U427
Flp-In™ CV-1	Thermo Fisher Scientific	RRID: CVCL_U425
Flp-In™ T-Rex™ HEK	Thermo Fisher Scientific	RRID: CVCL_U427

**Experimental models: Organisms/strains**		

Wistar-Han rats (Male)	Janvier Labs, France	RRID: RGD_38676310
C57BL6/J mice (Female)	Charles River, France	RRID: IMSR_JAX:000664

**Oligonucleotides**		

Select Negative Control siRNA	Ambion	Cat# 4390843
Select hamster Slc2a1 siRNA #1	Ambion	sense 5′-CCGCUAUGGAGAGCCCAUUtt-3’; antisense 5′-AAUGGGCUCUCCAUAGCGGtg-3′
Select hamster Slc2a1 siRNA #2	Ambion	sense 5′-GCUGCUCAGUAUCAUCUUCtt-3’; antisense 5′-GAAGAUGAUACUGAGCAGCag-3′
Gapdh	Microsynth	Fw 5′-ggatgcagggatgatgttct -3’; Rv 5′-ttgtgatgggtgtgaaccac-3’ (238bp)
Kcnc4	Microsynth	Fw 5′-cagaagcttcccaagaaacg-3’; Rv 5′-tagcgtcaccattctgcttg-3’ (184bp)
Slc2a1	Microsynth	Fw 5′-tgtcgctgttcgtggtagag-3’; Rv 5′-aagacataggggccacacag-3’ (334bp)
N257Q	Microsynth	5′-gccttcaacattgaccgaCAGgtgacggagatccacc-3’; 5′-ggtggatctccgtcacCTGtcggtcaatgttgaaggc-3′
N266Q	Microsynth	5′-gatccaccgggtagggCAGatcaccagcgtgcgcttc-3’; 5′-gaagcgcacgctggtgatCTGccctacccggtggatc-3′

**Software and algorithms**		

MATLAB	MathWorks	Version: R2024b
Lasergene	DNASTAR	Version: 17

**Other**		

Protease inhibitor cocktail tablets	Sigma-Aldrich	Cat# 11873580001
Nonidet P40	MP Biomedicals	Cat# 11RIST1315
Protein G Agarose	Roche	Cat# 11719416001
Sepharose 6B	Sigma-Aldrich	Cat# 6B100
Supported Nitrocellulose Membrane	BioRad	Cat# 1620094

### Experimental model and study participant details

#### Animal samples

Brain tissues were collected from the whole brain, cerebellum, or cerebral cortex of 6-month-old male Wistar-Han rats or 1-year-old female C57BL/6J mice. All animal procedures were carried out in agreement with the animal authorization (VD1550) approved by the Veterinary Authorities and the Cantonal Commission for Animal experimentation of the Canton of Vaud, according to the Swiss animal protection law.

### Method details

#### Cell line generation and maintenance

The Flp-In CHO (Chinese Hamster Ovary; RRID: CVCL_U427), Flp-In CV-1 (Cercopithecus aethiops; RRID: CVCL_U425) and Flp-In T-Rex HEK (Human Embryonic Kidney; (RRID: CVCL_U427) cell lines were purchased from Invitrogen-Life Technologies. The Flp-In CHO and Flp-In CV-1 were converted in Flp-In T-Rex as previously described (Ranjan et al., 2019) to generate isogenic tetracycline inducible cell lines. For simplicity, Flp-In T-Rex is abbreviated as FT. The CHO-FT-kv3.4, HEK-FT-kv3.4 and CV-1-FT-kv3.4 cell lines were generated as previously described (Ranjan et al., 2019, cell lines library construction), for an on-demand expression of the kv3.4 ion channel upon tetracycline induction (rat kcnc4: NM_001122776, mouse kcnc4: NM_145922 and human kcnc4: NM_004978). Cell handling and maintenance for mentioned cell lines are described in Ranjan et al., 2019.

#### Induction of ion channel expression and drug treatment

Cells are plated and grown for 24 h in their respective culture medium, free of antibiotics. The number of cells and the type of culture vessels are depending on the specific experiment. Ion channel expression is induced by adding 1 μg/μl tetracycline in the culture medium for 24 h. In case of treatment, cells are treated with the indicated drug or compound, in the culture medium at the time of tetracycline induction for 24 h. The different drugs and compounds, with their corresponding concentration used are listed in the material section.

#### Glucose deprivation and recovery procedures

To test the effect of glucose deprivation, cells are grown in glucose-deprived medium for 3 days before plating, induced with tetracycline 24 h after plating for another 24 h in same glucose deprived medium; the condition is mentioned as “low glucose” because a residual amount of glucose is present in the serum used to keep the cells alive (low glucose medium contains 0.04–0.09 g/L glucose versus 2 g/L in normal medium; the list of the different culture media used, with the corresponding concentration of glucose is listed in the material section). When a recovery test is performed, 5 mM D(+)Mannose, Sodium Pyruvate or BHB are added in low-glucose medium at the time of tetracycline induction of the ion channel (see material section for reference). In case of glucose recovery, cells are induced with tetracycline in their standard culture medium.

#### siRNA transfection

CHO cells are transfected with siRNA by electroporation using the Neon transfection system (Invitrogen). Briefly, cells at 80% confluence are detached with trypsin, washed with PBS, counted and resuspended in “Buffer R” at 1 × 10^7^ cells/ml. 0,5 million cells are mixed with 10 μL of 100 mM hamster Slc2a1 siRNA #1 and #2, or with 20 μL of 100 mM negative control siRNA in a final volume of 110 μL Buffer R. Cells are then electroporated following the manufacturer’s instructions using the following pulse program: “1500 V, 10 ms width, 3 pulses”. Cells are then displayed in a 10 cm culture plate pre-filled with 10 mL pre-warmed culture medium (200 nM final concentration of siRNA) for 48h before analysis. The references and sequences of siRNA are listed in the material section.

#### mRNA extraction and RT-PCR

RNA was isolated using the RNeasy Mini Kit (Qiagen), following the supplier’s protocol. First-strand cDNA was synthesized from 2 μg of total RNA using SuperScript III Reverse Transcriptase and oligodT, according to the manufacturer’s instructions, in a 20 μL final volume. The expression of the targeted gene is then checked by PCR. Briefly, 1 μL of the cDNA product is used for PCR amplification (a 1:50 dilution of the cDNA is used in case of overexpressed gene), using gene-specific primers on a thermal cycler (SensiQuest) with 28 cycles of amplification. The housekeeping gene *gapdh* is used as internal control. The sequences of the primers used are listed in the material section.

#### Gene mutagenesis

To introduce the targeted mutation on the Kv3.4 sequence, the GeneArt Site-Directed Mutagenesis kit (Invitrogen-Life Technologies) is used according to manufacturer’s instructions. Briefly, a PCR reaction using the PFU enzyme is performed on 20 ng of the plasmid containing the targeted gene, with two overlapping primers containing the target mutation. A recombination reaction is then performed to circularized the plasmid. The sample is transformed into DH5a-T1 competent E-coli, that digest the methylated template DNA, leaving only the mutated product. The transformants are analyzed by DNA electrophoresis and the presence of the mutation is confirmed by sequencing. Primers containing the desired mutation are designed using the DNASTAR Lasergene software SeqBuilder and listed in the material section. The double mutant Kv3.4_N257/266Q is generated by sequential mutagenesis.

#### Brain tissue collection

Brain tissues were collected from the whole brain, cerebellum or cerebral cortex of male Wistar-Han rats or female C57BL6/J mice. The brain tissues were collected in Qiazol Lysis reagent (Qiagen) in case of subsequent RNA isolation, or directly frozen at −80°C in case of subsequent protein extraction.

#### Immunoblotting (western-blot)

All western blots were performed in at least duplicate. Cells were seeded on 6 cm plates at 0.5 million cells per plate 24 h before induction and,or treatment. When indicated, control cells were left non-induced or untreated. Cells are then washed in PBS, collected by trypsinization and lysed in lysis buffer (50 mM Tris (pH 8), 150 mM NaCl, 1% NP40 and protease inhibitors) at 4°C. Samples are sonicated (3 × 1 sc pulses) before incubation on ice for 30 min. Lysates are then clarified by centrifugation at 12′000 rcf for 5 min at 4°C. Clear lysates are quantified using the Pierce 660 nm Protein assay. 3× Blue Loading Buffer-DTT (New England BioLabs) is added to 20 μg of the clear supernatant and solubilized proteins are boiled 5 min before separation by SDS-PAGE following standard procedures. Briefly, proteins are transferred to nitrocellulose membranes (BioRad) and blocked in TBS-T (TBS +0.2% Tween 20) supplemented with 4% non-fat dried milk powder (AppliChem). Membranes are incubated overnight at 4°C in TBS-T-milk with primary antibody, washed three times in TBS-T before 1h incubation with secondary antibody at room temperature. Finally, detection is performed using the PierceTM ECL Western Blotting Substrate (Thermo Fisher Scientific). The antibodies used with their concentration are listed in the material section.

#### Cell surface protein biotinylation

CHO cells (CHO-FT-Kv3.4, Kv3.4_N257Q, Kv3.4_N266Q and Kv3.4_N257/266Q) are seeded on four 10 cm plates at 1 million cells per plate, before induction. In case of treatment with tunicamycin, the drug is added at the time of tetracycline induction. Cell surface proteins are labeled with biotin and purified by using the PierceTM Cell Surface Protein Isolation kit, following the manufacturer’s instructions. At the end of the cell lysis step, 5% of the total protein is collected as “input” sample to use as positive control in subsequent immunoblotting (total lysate). Total lysate and cell surface biotinylated fraction (membrane protein) are then tested for kv3.4 expression by western-blot. The expression of the intracellular protein Actin is tested as control for purity of the membrane fraction. Figure 2F Lysate “input” panel of the Kv3.4 WB, the same sample was reloaded again for the blotting.

#### Membrane and cytoplasmic proteins separation

CHO-FT-Kv3.4 cells are plated at 1 million on a 10 cm plate, and induced for ion channel expression as described. Cells are washed in PBS, collected by trypsinization and subjected to the Mem-Per Plus Membrane Protein Extraction Kit (Thermo-scientific), following the manufacturer’s instructions to separate membrane and cytosolic fractions. Briefly, cell pellets are washed with “cell wash solution”, then resuspend in 700 mL of “permeabilization Buffer” supplemented with protease inhibitors, and incubated for 10 min at 4°C with constant mixing on a shaking platform. After 15 min high speed centrifugation at 4°C, the supernatant corresponding to the **cytosolic fraction** is collected. The remaining pellet is resuspended in 500 μL of “solubilization buffer” supplemented with protease inhibitors and incubated for 30 min at 4°C with constant mixing on a shaking platform. After 15 min high speed centrifugation at 4°C, the supernatant corresponding to the **membrane fraction** is collected. In case of brain tissue, 20–40 mg of tissues are mechanically homogenized in 700 μL of permeabilization Buffer supplemented with protease inhibitors using a plastic piston until an even suspension is obtained. The separation protocol is then performed as described before.

#### PNGase F and Endo H treatment

When indicated, 10–20 μg of protein lysates are treated with 1 μL PNGase F (New England BioLabs) or 1 μL Endo H (New England BioLabs) for 1 h at 37°C in a total volume of 20 μL, according to the manufacturer’s instructions.

#### Kv3.4 immunoprecipitation

CHO-FT-Kv3.4 cells are seeded on 10 cm plates at 1 million cells per plate, 24 h before induction with tetracycline. Cells are then washed in PBS, collected by trypsinization and lysed in 200 μL cold lysis buffer (50 mM Tris (pH 8), 150 mM NaCl, 1% NP40 and protease inhibitors). Samples are sonicated (3 × 1 sc pulses) before incubation on ice for 30 min. Lysates are then clarified by centrifugation at 12′000 rcf for 5 min at 4°C. 30 μL of lysates are left apart as total lysate for control. The 170 μL left are incubated with 10 μL of pre-binding antibody (see below) + 10 μL of Sepharose 6B overnight at 4°C on a rotator. The agarose beads are then washed 3 times with lysis buffer containing decreasing concentration of NP40 (1%, 0.1% and then 0.05%), resuspended in 25 μL of Reducing Blue Loading buffer (SDS-DTT 1×) and boiled 5 min at 100°C before analysis by western blot.

When indicated, solubilized membrane protein extracts are immunoprecipitated for Kv3.4 following the exact same protocol except that after washing, agarose beads are resuspended in 10 μL lysis buffer for treatment with 1 μL PNGase F (New England BioLabs) as described in the method section.

Before cell lysis, “pre-binding antibody” is prepared incubating 10 μL of protein G Agarose (previously washed and resuspended in lysis buffer) with 1 μL of the anti-Kv3.4-N-terminus antibody (Neuromab), for 1 h incubation on ice with regular gentle handshaking (every 10–15 min). An anti-Kv3.4-C-terminus antibody is then used for the detection of kv3.4 after immunoprecipitation by western-blot.

#### Lectin binding assay

CHO-FT-Kv3.4 cells are seeded on 6 cm plates at 0,5 million cells per plate, 24h before induction with tetracycline. After 24h, cells are washed with cold PBS containing CaCl_2_ and MgCl_2_ (PBS^+^,Thermo Fisher Scientific), before 10 min incubation at 4°C in the same PBS^+^. Cells are further incubated 10 more min at 4°C in 2 mL PBS^+^ containing biotinylated lectin (20ug ConA, 4ug RCA or 4ug WGA; see material for details). After incubation, cells are washed once in PBS^+^, gently scrapped in 2 mL PBS^+^ and centrifuged at 800 rcf for 3 min. Supernatant is discarded, and cells are resuspended in 120 μL Triton Lysis Buffer (PBS, 1 mM CaCl_2_, 1 mM MgCl_2_, 1% Triton, protease inhibitors), for 20 min on ice. The lysate is centrifuged 10 min at 14,000 rcf at 4°C. 20 μL of the clear lysate is left apart as total lysate for control. The 100 μL clear lysate left is incubated with 10 μL streptavidin-coupled beads (Dynabeads MyOne streptavidin C1, Invitrogen-Life Technologies) for 2h at 4°C on a rotator. Beads are then washed three times with PBS and resuspended in 20 μL 1× Blue Loading Buffer-DTT and boiled before analysis by immunoblotting.

#### Overlay plot and box-plot

The current trace elicited in response to an activation stimulus at +80 mV has been normalized to the peak current for each cell. The overlay plot displays these normalized current traces from various cells, spanning from 70 ms to 599 ms. Inactivation width (Inac-width) is defined as the width at 70% height of this normalized current trace. The distribution of Inac-widths is presented using a boxplot, which depicts the median and quartiles (1^st^ and 3^rd^). The distance between the bottom and top of each box is the interquartile range. The whiskers are lines extending above and below each box. Whiskers go from the end of the interquartile range to the furthest observation within the whisker length. Observations beyond the whisker length are marked as outliers. In our analysis, an outlier is a value that is more than 1.5 times the interquartile range away from the bottom or top of the box. Heterogeneity in inactivation kinetics is represented by the spread between the whiskers of the boxplot. Absolute values of respective whisker ranges are available in Table S1.

#### Manual patch clamp

Manual patch-clamp experiments were carried out on rat CHO Kv3.4 cell line. Cells were plated on 18 mm diameter coverslips coated with Poly-L-Lysine (50ug/ml) at room temperature. Cells were visualized by infrared differential interference contrast video microscopy utilizing a CCD camera (PCO Imaging) mounted on an upright microscope (LNscope Luigs & Neumann GMBH, Germany). Cells were continuously perfused with artificial cerebrospinal fluid (ACSF) containing (in mM): 125 NaCl, 25 NaHCO_3_, 2.5 KCl, 1.25 NaH_2_PO_4_, 2 CaCl_2_, 1 MgCl_2_ and 25 D-glucose bubbled with 95% O_2_ and 5% CO_2_. The intracellular pipette solution contained (in mM) 110 potassium gluconate, 10 KCl, 4 ATP-Mg, 10 phosphocreatine, 0.3 GTP, 10 HEPES adjusted to pH 7.3–7.4 with 5 M KOH. Osmolarity was adjusted to 290–300 mOsmol with D-mannitol. The whole-cell recordings were performed in a temperature controlled mini chamber with Multiclamp 700B amplifiers in the voltage-clamp mode at 25°C and 37°C. The recording temperature was controlled with LN Temperature controller VII (Luigs & Neumann GMBH, Germany). Data acquisition was performed via ITC-1600 board, connected to a PC running a custom-written routine under IGOR Pro 7 (Wave metrics, Portland, USA). Sampling rates were 10 KHz and the current signal was filtered with a 2 kHz Bessel filter. Patch pipettes were pulled with a Flaming/Brown micro-pipette puller P-97 (Sutter Instruments Co, Novato, CA, USA) of a DMZ puller (Zeitz Instruments, Martinsried, Germany) and had an initial resistance of 5–8 MΩ.

#### Automated patch clamp

Automated patch clamp experiments were carried out as described before (Ranjan et al. 2019). In summary, a dedicated 2.5 m × 2.5 m temperature-controlled room was used to record cells in whole-cell patch clamp configuration on Nanion’s NPC-16 Patchliner Quattro (Nanion Technologies GmbH) fitted with EPC-10 HEKA Quadro amplifiers (HEKA Electronik GmbH). Disposable borosilicate (Nanion NPC-16) medium resistance (2–3 MΩ) glass chips were used for all recordings. The PatchControlHT software (Nanion Technologies GmbH) was used for the automation of patch clamp steps (cell capture, seal formation, whole-cell access and washing, etc.). The PatchControlHT software internally uses Patchmaster software (HEKA) for the data acquisition. The data were filtered with internal filter 1 (Bessel) at 10 kHz and filter 2 (I_Bessel) at 2.9 kHz of the EPC-1 10 and digitized at 10–50 KHz configuration according to different voltage protocols. The extracellular solution (ECS) contained: 140 mM NaCl, 4 mM KCl, 1 mM MgCl2, 2 mM CaCl2, 5 mM D-Glucose monohydrate, 10 mM HEPES; pH 7.4 with NaOH, osmolarity 298 mOsm. The intracellular solution (ICS) contained: 50 mM KCl, 10 mM NaCl, 60 mM K-Fluoride, 20 mM EGTA, 10 mM HEPES; pH 7.2 with KOH, osmolarity 285 mOsm. The Seal enhancer solution (SES) contained: 80 mM NaCl, 3 mM KCl, 10 mM MgCl2, 35 mM CaCl2, 10 mM HEPES (Na+salt); pH 7.4 with HCl, osmolarity 298 mOsm. All solutions were filtered with 0.2 μm filter (GP ExpressPLUS membrane, Millipore) and stored in 50 mL aliquots at +4 °C.

#### Cell selection criteria for electrophysiology data

The first phase of cell selection for electrophysiology data involved evaluating key recording parameters: voltage offset (V-offset), whole-cell seal resistance (post whole-cell configuration), series resistance (R-Series), and slow capacitance (C-slow). The preliminary acceptance criteria were as follows: V-offset <45 mV, seal resistance >200 MΩ, R-Series <15 MΩ, and C-slow <35 pF. In the second stage of filtering, a maximum current amplitude criterion was applied, selecting cells with currents between 0.5 nA and 45 nA.

These criteria effectively eliminated the majority of poor-quality recordings. However, in some cases, cells that met all these parameters still exhibited unstable membrane currents, which could not be identified solely through standard electrophysiological metrics. Such cells were manually reviewed and excluded from the final analysis.

### Quantification and statistical analysis

Statistical analyses were performed using custom MATLAB scripts (Version R2024b). Inac-width distributions are shown as boxplots depicting the median and the 1st and 3rd quartiles. Whiskers on the box indicate variability outside the upper and lower quartiles. Heterogeneity in inactivation kinetics (Figures 1, 2, 3, 5, S3, S4, and S6) is represented by the spread between whiskers. The number of cells analyzed for each condition is provided in Table S1. Pearson’s correlation coefficients between Kv3.4 maximum current and inactivation width (Figure S2) were calculated and plotted using MATLAB’s corrplot function.
