# Supplementary material for: N-glycosylation modulates the inactivation kinetics of the Kv3.4 ion channel
Source: iScience. 2025 Aug 21;28(9):113409. doi: 10.1016/j.isci.2025.113409 (PMC12455010; doi:10.1016/j.isci.2025.113409)
Supplement: Document S1. Figures S1–S6 and Tables S1 and S2 [file mmc1.pdf]

iScience, Volume *28*

Supplemental information

N-glycosylation modulates the inactivation kinetics of the Kv3.4 ion channel

Rajnish Ranjan, Emmanuelle Logette, Mirjia Herzog, Valerie Buchillier, Enrico Scantamburlo, and Henry Markram

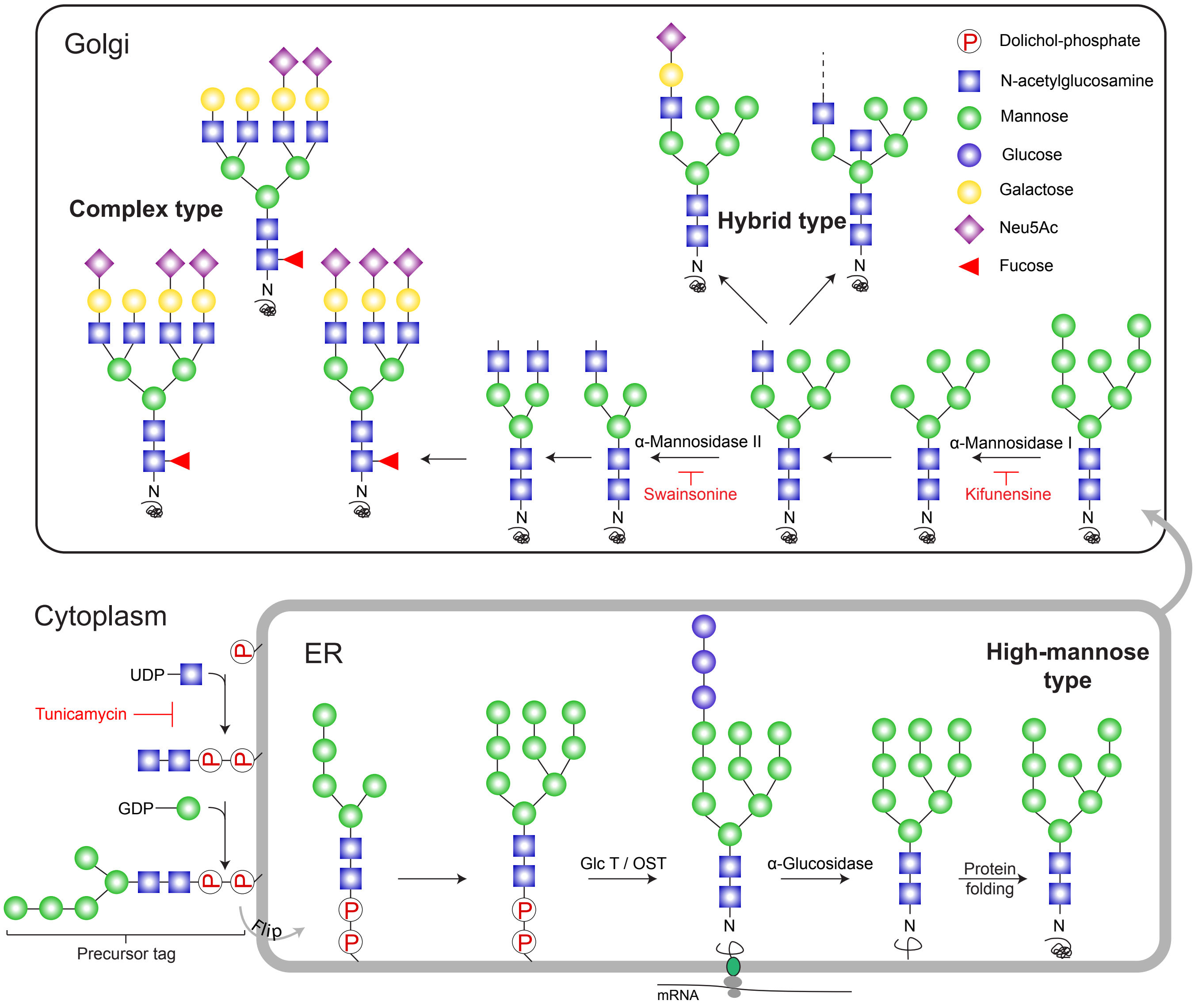

Figure S1: Overview of the N-glycosylation pathway
Schematic representation of the main enzymatic steps in the N-glycosylation pathway, adapted from Vasconcelos-dos-Santos et al., Front. Oncol. (2015). Glycosylation begins in the cytoplasmic side of the ER with the assembly of a dolichol-linked precursor oligosaccharide composed of N-acetylglucosamine and mannose. After flipping into the ER lumen, glucose residues are added by glucose transferase (Glc T), and the full glycan is transferred co-translationally to the nascent protein by oligosaccharyltransferase (OST). Initial trimming of glucose residues by α-glucosidase enables proper protein folding, resulting in high-mannose glycoforms. Further processing in the Golgi by α-mannosidase I and II yields hybrid- and complex-type N-glycans. Drug targets used in this study are indicated: tunicamycin inhibits precursor synthesis, kifunensine blocks α-mannosidase I (preventing hybrid/complex maturation), and swainsonine inhibits α-mannosidase II (blocking complex glycan formation)

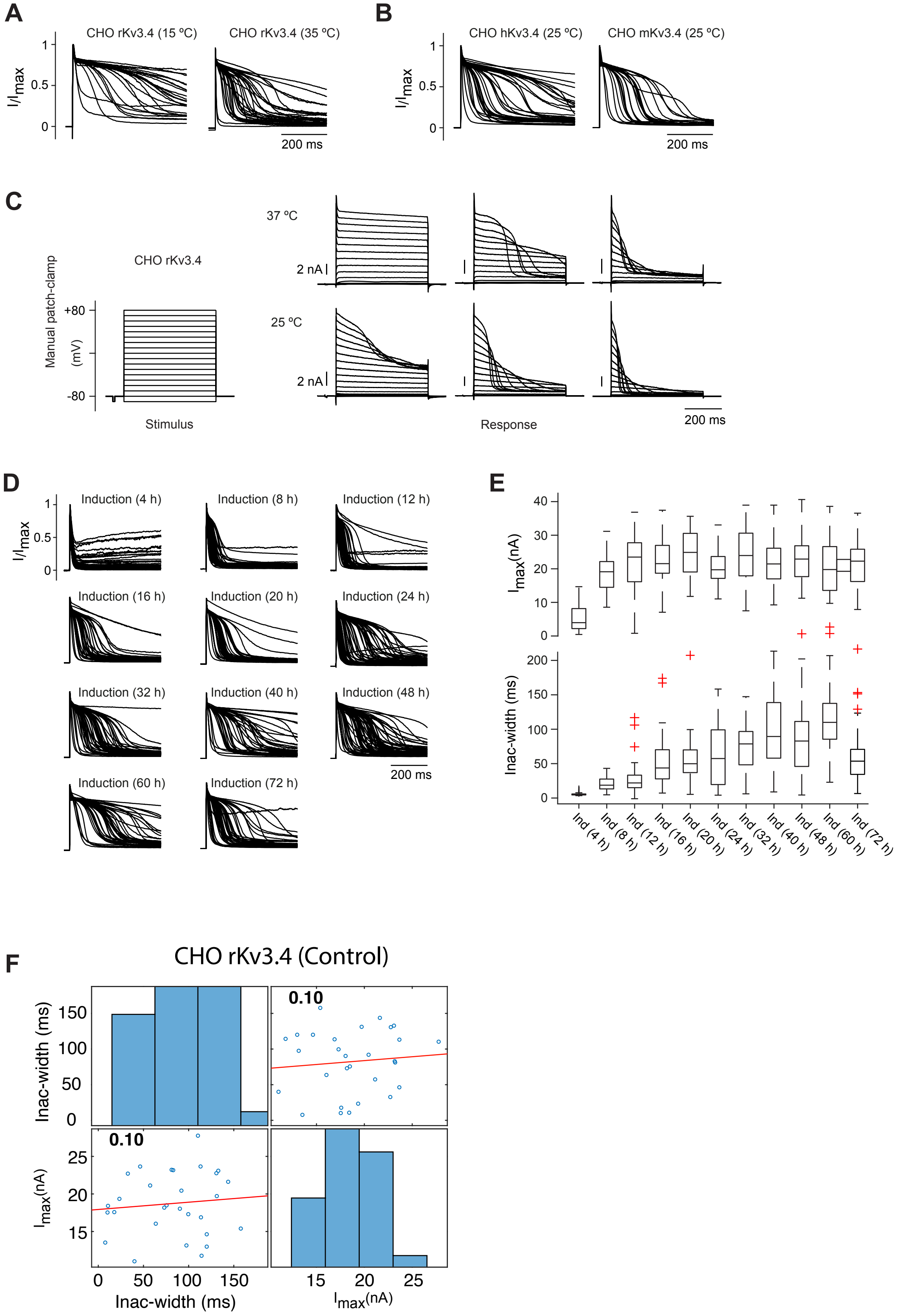

Figure S2: Heterogeneous inactivation delay of Kv3.4 channels across conditions and species
(A) Overlay plot of evoked CHO rKv3.4 current traces recorded with an APC at 15°C (n=25) and 35°C (n=34), normalized to each cell's maximum current (I_{max}). **(B)** Overlay of normalized current traces for CHO cells expressing human (hKv3.4, n=35) and mouse Kv3.4 (mKv3.4, n=23) recorded at 25°C **(C)** Representative traces from manual patch clamp recordings of CHO rKv3.4 cells at 25°C and 37°C in response to the activation protocol. **(D)** Overlay plots of the current traces illustrate heterogeneity in inactivation delay after induction duration ranging from 4 hours to 72 hours. **(E)** Maximum current (I_{max}) and kinetic heterogeneity for each group (Induction 4 h, n = 36; 8 h, n = 36; 12 h, n = 36; 16 h, n = 40; 20 h, n = 37; 24 h, n = 59; 32 h, n = 47; 40 h, n = 40; 48 h, n = 64; 60 h, n = 38; 72 h, n = 42) is quantified by the box plot of I_{max} and inactivation width (inac-width). **(F)** Scatter plot showing lack of correlation (Pearson's r=0.1) between I_{max} and Inac-width in cells analyzed in Figure 1D, suggesting that kinetic variability is not dependent on current amplitude.

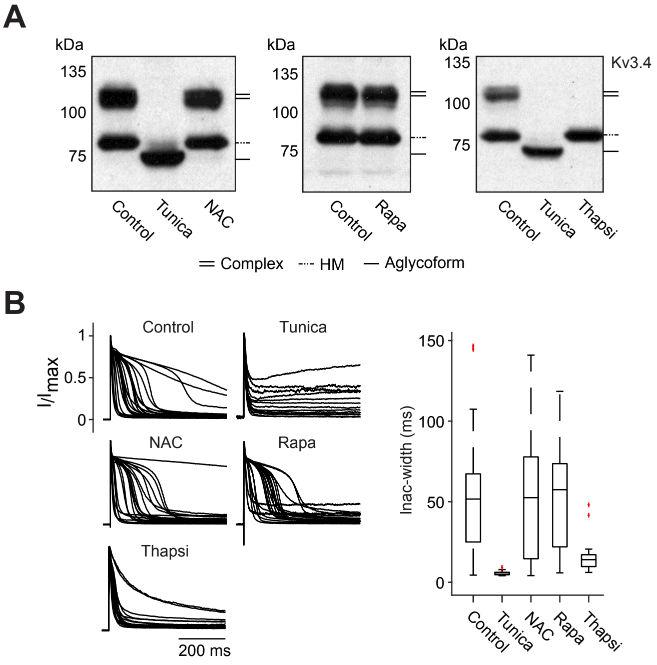

Figure S3: Impact of N-glycosylation disruption on Kv3.4 glycosylation and kinetic heterogeneity

(A) Western blot analysis of CHO rKv3.4 cells after treatment with different compounds: tunicamycin (Tunica), N-acetylcysteine (NAC), rapamycin (Rapa), and thapsigargin (Thapsi). Western blotting was performed using an anti-Kv3.4 C-terminal anitbody. Complex-glycosylated, high-mannose and aglycosylated Kv3.4 bands are shown **(B)** Kinetic heterogeneity in inactivation delay is shown by overlay plots and quantified using box plots (Control, n = 23; Tunica, n = 13; NAC, n=20; Rapa, n=20; Thapsi, n=16).

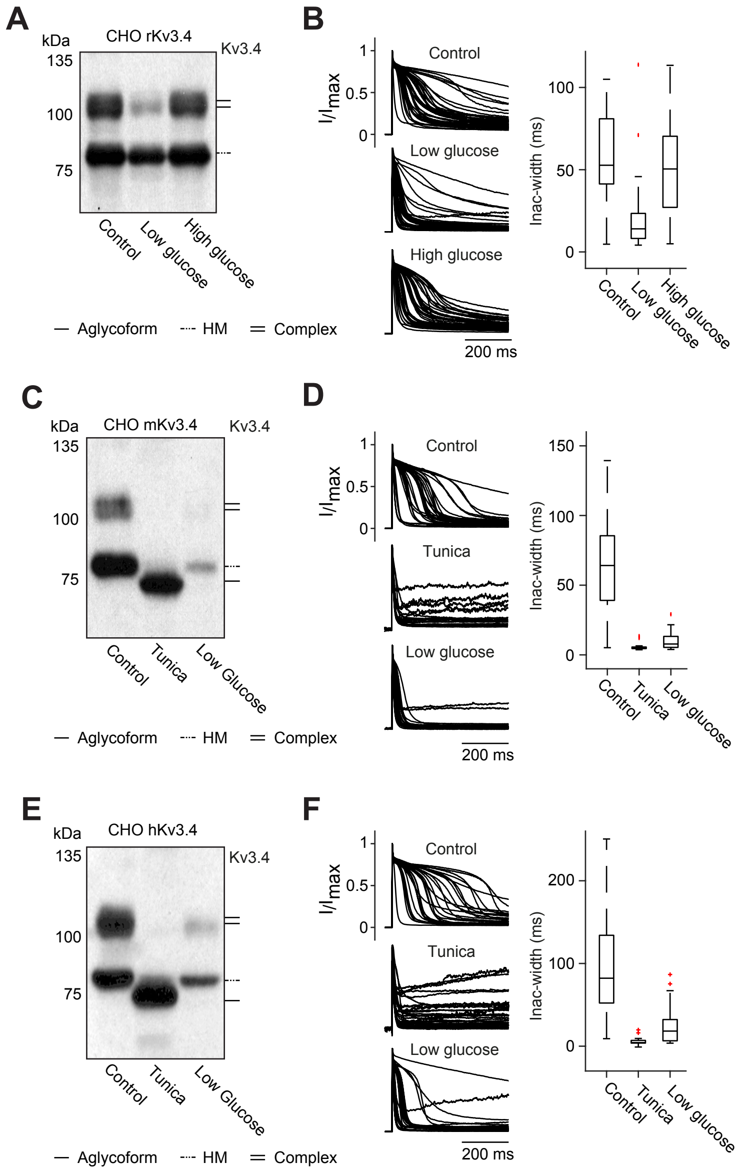

Figure S4: Glucose concentration affects the N-glycosylation and inactivation delay kinetics of Kv3.4
(A) Western blot analysis of CHO cells expressing rat Kv3.4 (rKv3.4) cultured in presence of different glucose concentrations. **(B)** Heterogeneity in Inactivation delay is shown with overlay plots and quantified using box plots (Control, n=38; Low glucose, n=33; High glucose, n=38). **(C and E)** Western blot analysis of CHO cells expressing mouse Kv3.4 (mKv3.4) or human Kv3.4 (hKv3.4) under control conditions, after tunicamycin treatment, or cultured in low glucose medium. **(D and F)** Overlay plots and box plots illustrate kinetic heterogeneity in each condition. (Mouse Kv3.4: Control, n = 30; Tunica, n = 22; Low glucose, n = 25; Human Kv3.4: Control, n = 29; Tunica, n = 32; Low glucose, n = 24). All western blotting was performed using an anti-Kv3.4 C-terminal antibody.

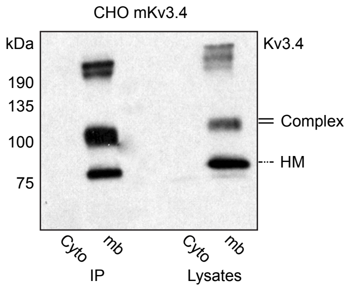

Figure S5: Membrane isolation and immunoprecipitation of Kv3.4
Membrane (mb) and cytoplasmic (Cyto) fractions were isolated from CHO cells expressing mouse Kv3.4 (mKv3.4), followed by immunoprecipitation using N-terminal Kv3.4 antibody and western blot analysis. Kv3.4 expression was detected exclusively in the membrane fraction, with no signal observed in the cytosol. All glycoforms of mKv3.4 were successfully immunoprecipitated from the membrane fraction. Western blotting was performed using an anti-Kv3.4 C-terminal antibody.

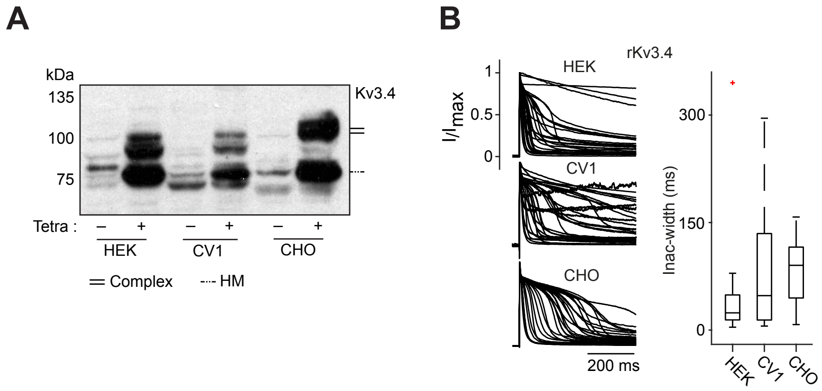

Figure S6: Cell line-specific glycosylation patterns of and kinetic heterogeneity of rKv3.4

Rat Kv3.4 (rKv3.4) was expressed in three different cell lines: HEK, CV1 and CHO. **(A)** Western blot analysis showing the glycosylation patterns of rKv3.4 across different cell lines. **(B)** The kinetic heterogeneity of rKv3.4 in each case is illustrated using overlay plots and quantified with box plots. (HEK, n = 31; CV1, n = 31; CHO, n = 29). Western blotting was performed using an anti-Kv3.4 C-terminal antibody

Figure	Panel	Plotted group	N	Whisker range(ms)	Median (ms)
**Figure 1**	**D**	**CHO rKv3.4**	29	150.04	90.3
**Figure 2**	**E**	**CHO rKv3.4 control**	20	140.92	72.27
		**CHO rKv3.4 + tunicamycin**	19	3.41	4.69
	**G**	**CHO rKv3.4 N257Q**	23	19.64	7.38
		**CHO rKv3.4 N266Q**	16	1.08	4.41
	**I**	**CHO rKv3.4 control**	29	150.04	90.3
		**CHO rKv3.4 + kifunensine**	29	79.39	27.51
		**CHO rKv3.4 + swainsonine**	29	112.77	60.8
**Figure 3**	**B**	**CHO rKv3.4 control**	29	137.51	56.41
		**CHO rKv3.4 + cytochalasin B**	19	40.75	18.85
	**D**	**CHO rKv3.4 sineg**	26	142.79	53.43
		**CHO rKv3.4 siGLUT1**	25	51.43	23.24
	**F**	**CHO rKv3.4 control**	41	117.36	50.99
		**CHO rKv3.4 low glucose**	39	74.59	21.255
		**CHO rKv3.4 low glucose + glucose recov**	41	96.97	39.486
		**CHO rKv3.4 low glucose + pyruvate reco**	27	21.58	9.93
		**CHO rKv3.4 low glucose + mannose reco**	35	96.83	43.254
		**CHO rKv3.4 low glucose + BHB recovery**	23	66.68	21.93

Figure 5

**A1**	**CHO rKv1.5 control**	29	468.99	107.43
	**CHO rKv1.5 + tunica**	19	91.68	52.81
	**CHO rKv1.5 + low glucose**	19	138.43	34.9
**B1**	**CHO rKv1.3 control**	43	307.78	82.36
	**CHO rKv1.3 + tunica**	36	95.39	45.74
	**CHO rKv1.3 + low glucose**	43	178.97	59.41
**C1**	**CHO rKv1.4 control**	21	13.4	14.13
	**CHO rKv1.4 + tunica**	22	5.61	13.03
	**CHO rKv1.4 + low glucose**	18	5.74	12.37
**D1**	**CHO rKv2.1 control**	19	269.86	356.17
	**CHO rKv2.1 + tunica**	18	259.96	438.42
	**CHO rKv2.1 + low glucose**	30	342.64	294.51

Figure S2

**A**	**CHO rKv3.4 (15 ℃)**	25	362.42	190.68
	**CHO rKv3.4 (35 ℃)**	34	141.05	51.83
**B**	**CHO hKv3.4**	35	236.38	82.52
	**CHO mKv3.4**	23	108.04	70.18
**E**	**CHO rKv3.4 (Ind 4h)**	36	4.24	4.98
	**CHO rKv3.4 (Ind 8h)**	36	38.46	18.66
	**CHO rKv3.4 (Ind 12h)**	36	52.41	21.17
	**CHO rKv3.4 (Ind 16h)**	40	102.07	43.11
	**CHO rKv3.4 (Ind 20h)**	37	94.12	49.62

		**CHO rKv3.4 (Ind 24h)**	59	154.2	57.4
		**CHO rKv3.4 (Ind 32h)**	47	141.2	75.74
		**CHO rKv3.4 (Ind 40h)**	40	204.55	82.91
		**CHO rKv3.4 (Ind 48h)**	64	197.76	82.37
		**CHO rKv3.4 (Ind 60h)**	38	184.05	105.07
		**CHO rKv3.4 (Ind 72h)**	42	116.85	50.03
**Figure S3**	**B**	**CHO rKv3.4 control**	23	102.96	42.78
		**CHO rKv3.4 + tunicamycin**	13	3.8	5.03
		**CHO rKv3.4 + NAC**	20	136.79	51.31
		**CHO rKv3.4 + rapamycin**	20	112.6	57.46
		**CHO rKv3.4 + thapsigargine**	16	14.48	12.4
**Figure S4**	**B**	**CHO rKv3.4 control**	38	100.27	50.68
		**CHO rKv3.4 low glucose**	33	41.66	13.13
		**CHO rKv3.4 high glucose**	38	108.5	50.44
	**D**	**CHO mKv3.4 control**	30	134.25	64.11
		**CHO mKv3.4 + tunicamycin**	22	2.8	4.77
		**CHO mKv3.4 low glucose**	25	17.73	7.44
	**F**	**CHO hKv3.4 control**	29	241.43	82.18
		**CHO hKv3.4 + tunicamycin**	32	10.34	4.22
		**CHO hKv3.4 low glucose**	24	63.45	10.14
**Figure S6**	**B**	**HEK rat Kv3.4**	31	75.24	20.75
		**CV1 rat Kv3.4**	31	290.02	43.07
		**CHO rat Kv3.4**	29	150.04	90.3

Table S1: Quantification of Kv3.4 inactivation kinetics heterogeneity, Related to STAR Methods

This table provides the exact whisker range values for the box-plot analysis of inactivation width, corresponding to all overlay plots discussed in this study. Additionally, median values and the number of cells analyzed for each case are also specified.

CHO-FT WT cells

	Prot_Name	Gene_Name	FPKM	transporter for :
**Glucose transport**	**GLUT1**	**Slc2a1**	**40.7**	**glc, gal, man, glucosamine**
	GLUT8	Slc2a8	4.59	glc, fru, gal
	GLUT13	Slc2a13	8.92	Does not transport glucose
	GLUT12	Slc2a12	0.0	-
	GLUT2	Slc2a2	0.0	-
	GLUT4	Slc2a4	0.4	-
	GLUT5	Slc2a5	0.0	-
	GLUT6	Slc2a6	0.1	-
	GLUT7	Slc2a7	0.0	-
	GLUT9	Slc2a9	0.0	-
**Monocarboxylate transport**	**MCT1**	**Slc16a1**	**102.5**	**lactate, pyruvate, ketone bodies**
	MCT7	Slc16a6	3	lactate, pyruvate, ketone bodies
	MCT11	Slc16a11	1.0	-
	MCT2	Slc16a7	0.6	-
	MCT13	Slc16a13	0.2	-
	MCT5	Slc16a4	0.1	-
	MCT4	Slc16a3	0.1	-
	MCT9	Slc16a9	0.0	-
	MCT10	Slc16a10	0.0	-
	MCT8	Slc16a2	0.0	-
	MCT5	Slc16a5	0.0	-
	MCT3	Slc16a8	0.0	-

Table S2: Expression profiles of GLUT and MCT transporters in CHO cells

This table presents transcriptomic data on endogenous expression of glucose (GLUT) and monocarboxylate (MCT) transporter genes in CHO-FT wild-type cells. Data are expressed as FPKM (Fragments Per Kilobase of transcript per Million mapped reads) and were obtained from the study by Ranjan et al. (2019). The table also includes the known transport specificity for each protein, providing functional context for their expression levels.
